# Supplementary material for: The microbiome of the Melitaea cinxia butterfly shows marked variation but is only little explained by the traits of the butterfly or its host plant
Source: Environ Microbiol. 2019 Sep 9;21(11):4253–69. doi: 10.1111/1462-2920.14786 (PMC6900084; doi:10.1111/1462-2920.14786)
Supplement: Supplementary file 2 — Supplementary Figure S1. Results of Dirichlet‐multinomial analysis. Panels A and B depict the Laplace goodness‐of‐fit measures for fitted mixture models with different number of mixture components (lower values corresponds to better fit). Panel C visualize the 2 mixture components of the best model for microbial OTUs in caterpillars. Supplement Fig. S2. Results of permutation tests. Each panel depicts the Spearman rank correlation coefficient between the assigned mixture component of the best 2‐component Dirichlet‐multinomial model for caterpillar OTUs data and available predictors. Red line corresponds to the real value and the black curve depict the density of permutation‐based values. Dashed blue lines depict the 2.5% and 97.5% quantiles of the permutation‐based density. Supplement Fig. S3. Principal Component Analysis (PCA) of the metabolites associated with the host plant. The PCA plot represents the ordination of the plant metabolites on the three first Principal Components (A) PC1 and PC2, (B) PC1 and PC3, (C) PC2 and PC3. The signal corresponding to the chemical shift of carbohydrates and amino acid residues are coloured in red while other signals are coloured in blue. Supplement Fig. S4. Partitioning of the explained variance of bacterial OTUs among the fixed and random effects in plant models. The coloured bars show, for each OTU, the proportions of variance attributed to each of explanatory variables. The average variance proportions over the OTUs are shown in the legend box. The ordering of OTU is following ordering of Fig. 1 except for the OTUs that were recorded only in larvae samples (for details, see Supplementary Table S2). See Statistical Methods for a full description of the included fixed and random effects. Supplement Fig. S5. The influence of metabolic covariates on plant microbiota. Regression coefficients that were estimated to be positive (respectively, negative) with 95% credibility level are shown by red (respectively, blue). The ordering [file EMI-21-4253-s002.pdf]

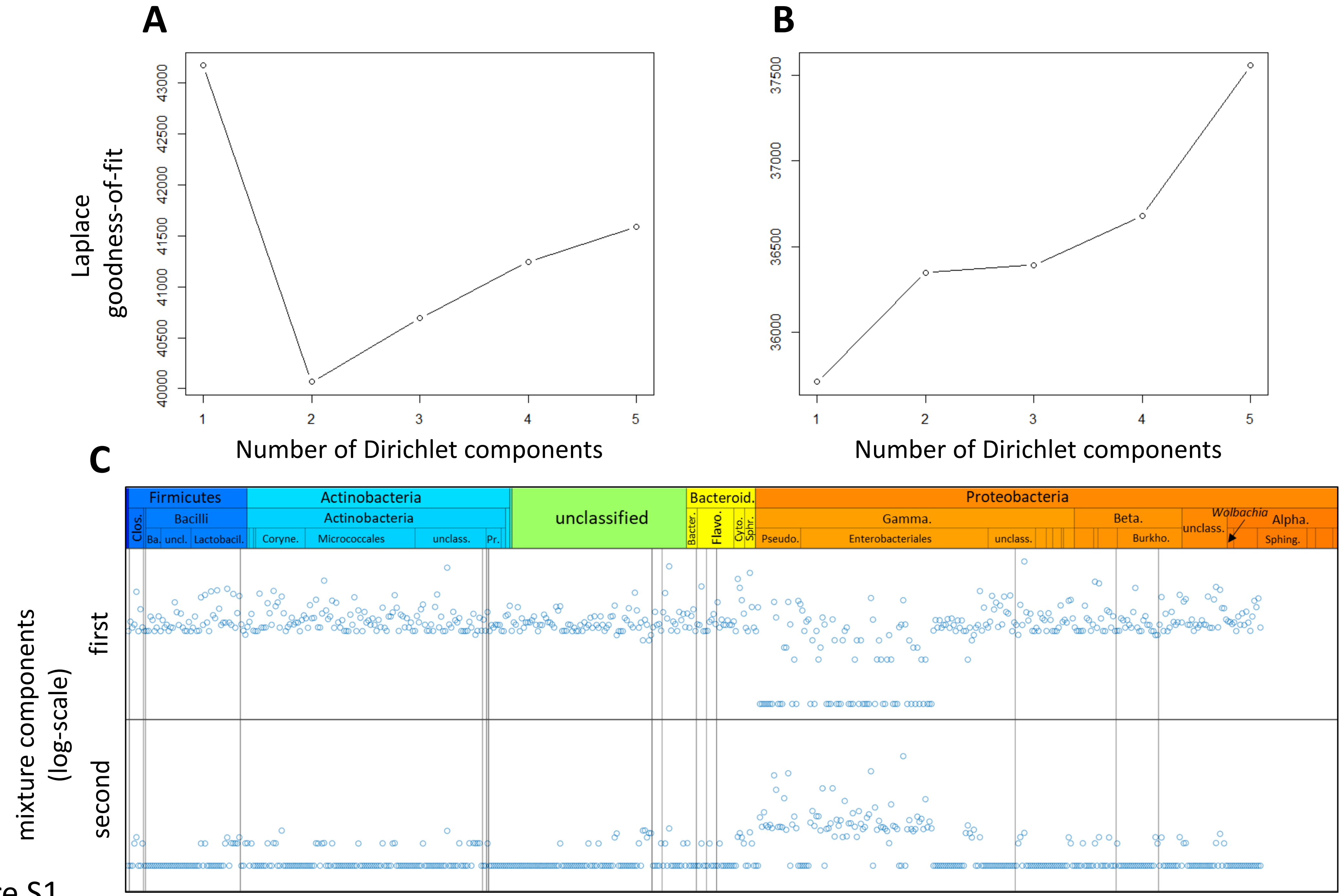
A
B
Laplace goodness-of-fit
Number of Dirichlet components
C
Firmicutes
Actinobacteria
unclassified
Bacteroid.
Proteobacteria
Clos.
Bacilli
Ba.
uncl.
Lactobacil.
Coryne.
Micrococcales
unclass.
Pr.
Bacter.
Flavo.
Cyto.
Sphr.
Gamma.
Pseudo.
Enterobacteriales
Beta.
Burkho.
Wolbachia
Alpha.
Sphing.
mixture components (log-scale)
first
second

Figure S1

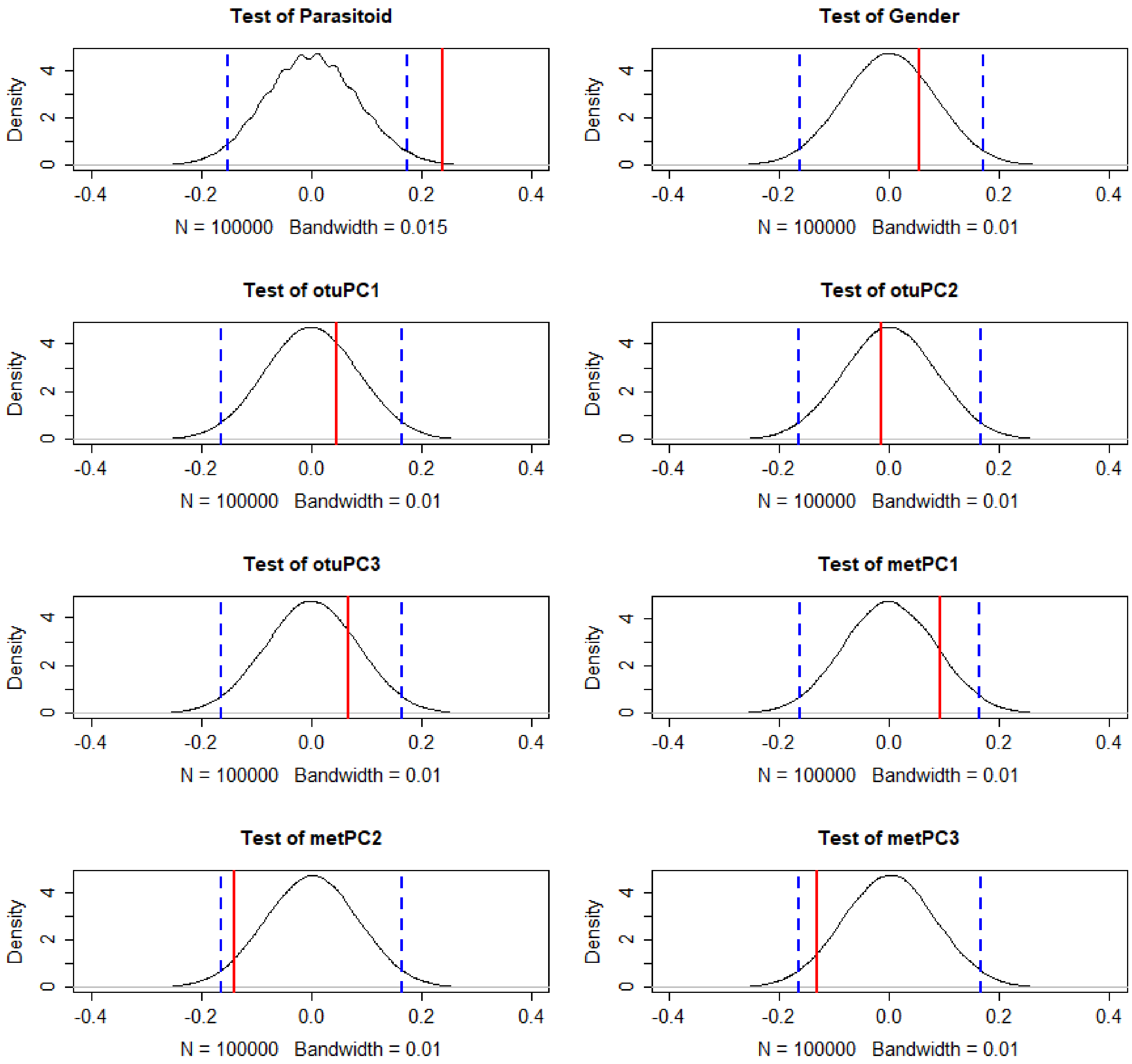
Test of Parasitoid
Density
N = 100000 Bandwidth = 0.015
Test of Gender
Density
N = 100000 Bandwidth = 0.01
Test of otuPC1
Density
N = 100000 Bandwidth = 0.01
Test of otuPC2
Density
N = 100000 Bandwidth = 0.01
Test of otuPC3
Density
N = 100000 Bandwidth = 0.01
Test of metPC1
Density
N = 100000 Bandwidth = 0.01
Test of metPC2
Density
N = 100000 Bandwidth = 0.01
Test of metPC3
Density
N = 100000 Bandwidth = 0.01

Figure S2

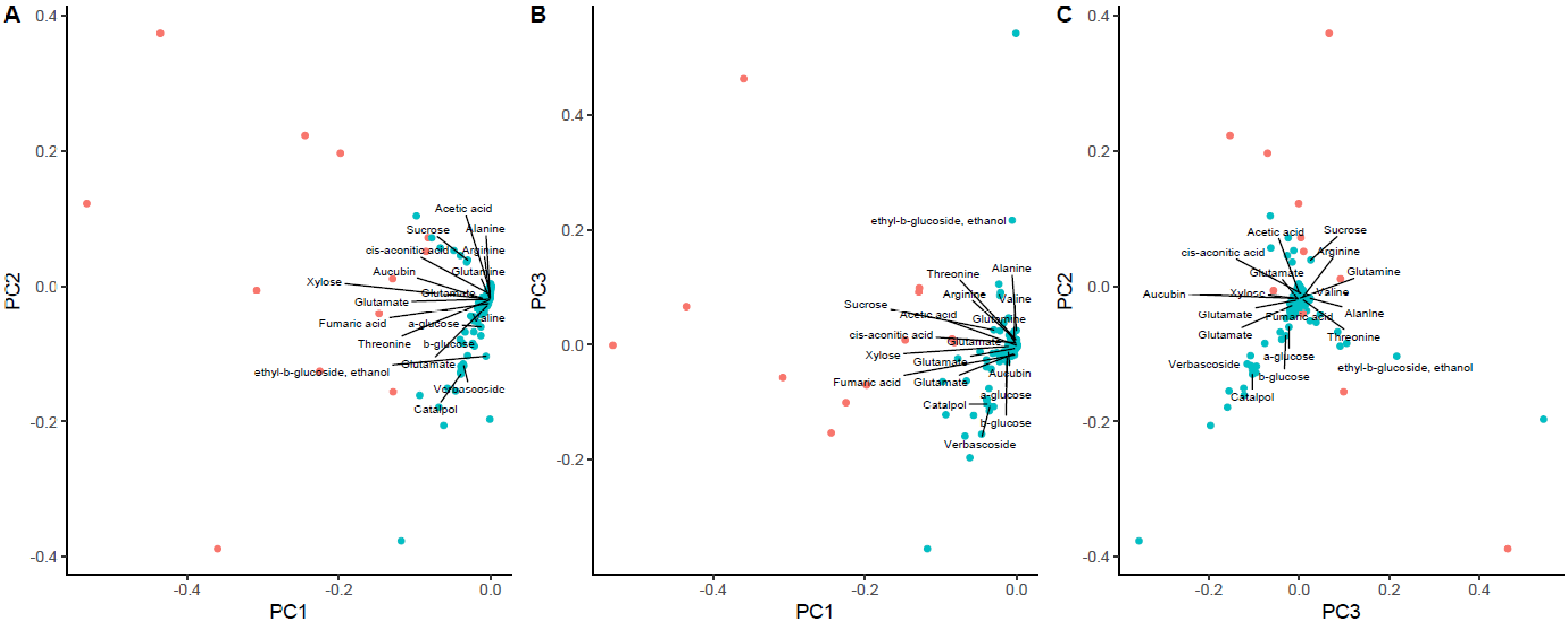
A
B
C
PC1
PC2
PC3
Acetic acid
Sucrose
Alanine
cis-aconitic acid
Arginine
Aucubin
Glutamine
Xylose
Glutamate
Valine
Fumaric acid
a-glucose
Threonine
b-glucose
ethyl-b-glucoside, ethanol
Verbascoside
Catalpol

Figure S3

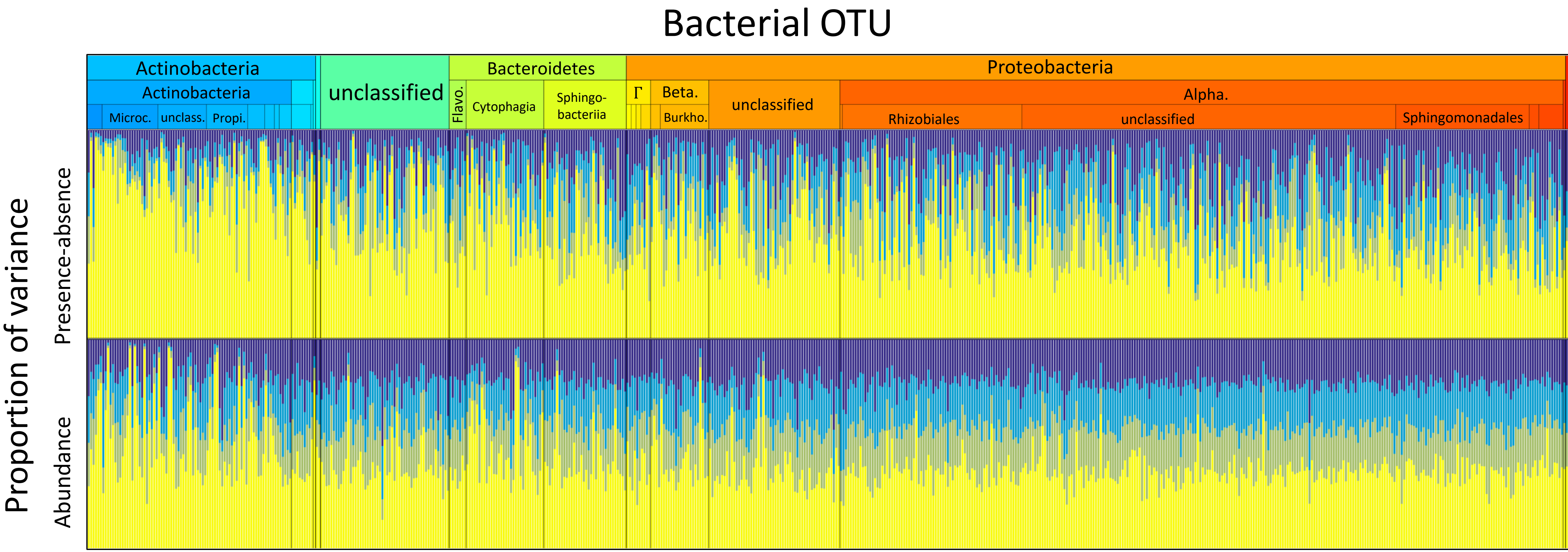

Variable	Type	Color	P-A (%)	Ab (%)
Plant metabolic composition (PC1)	Fixed effect		18	21
Plant metabolic composition (PC2)	Fixed effect		17	20
Plant metabolic composition (PC3)	Fixed effect		14	19
Plant level	Random effect		51	40

Figure S4

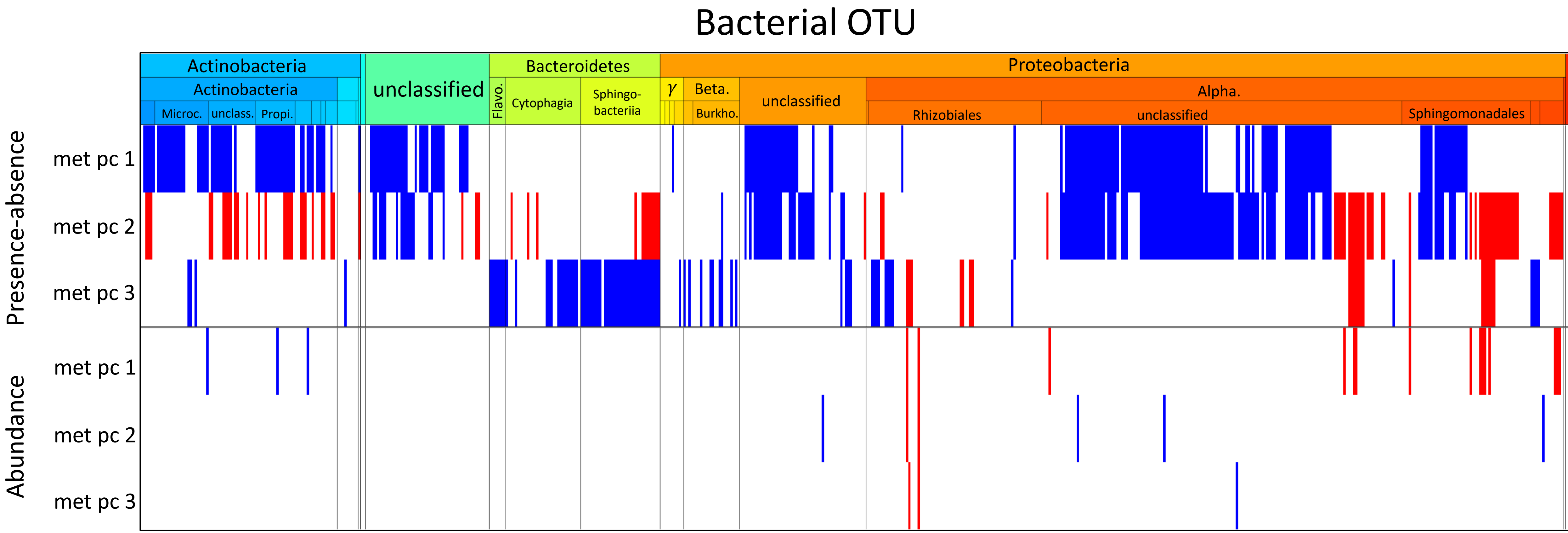
Bacterial OTU
Actinobacteria
Actinobacteria
Microc.
unclass.
Propi.
unclassified
Bacteroidetes
Flavo.
Cytophagia
Sphingo-
bacteriia
Proteobacteria
γ
Beta.
Burkho.
unclassified
Alpha.
Rhizobiales
unclassified
Sphingomonadales
Presence-absence
met pc 1
met pc 2
met pc 3
Abundance
met pc 1
met pc 2
met pc 3

Figure S5

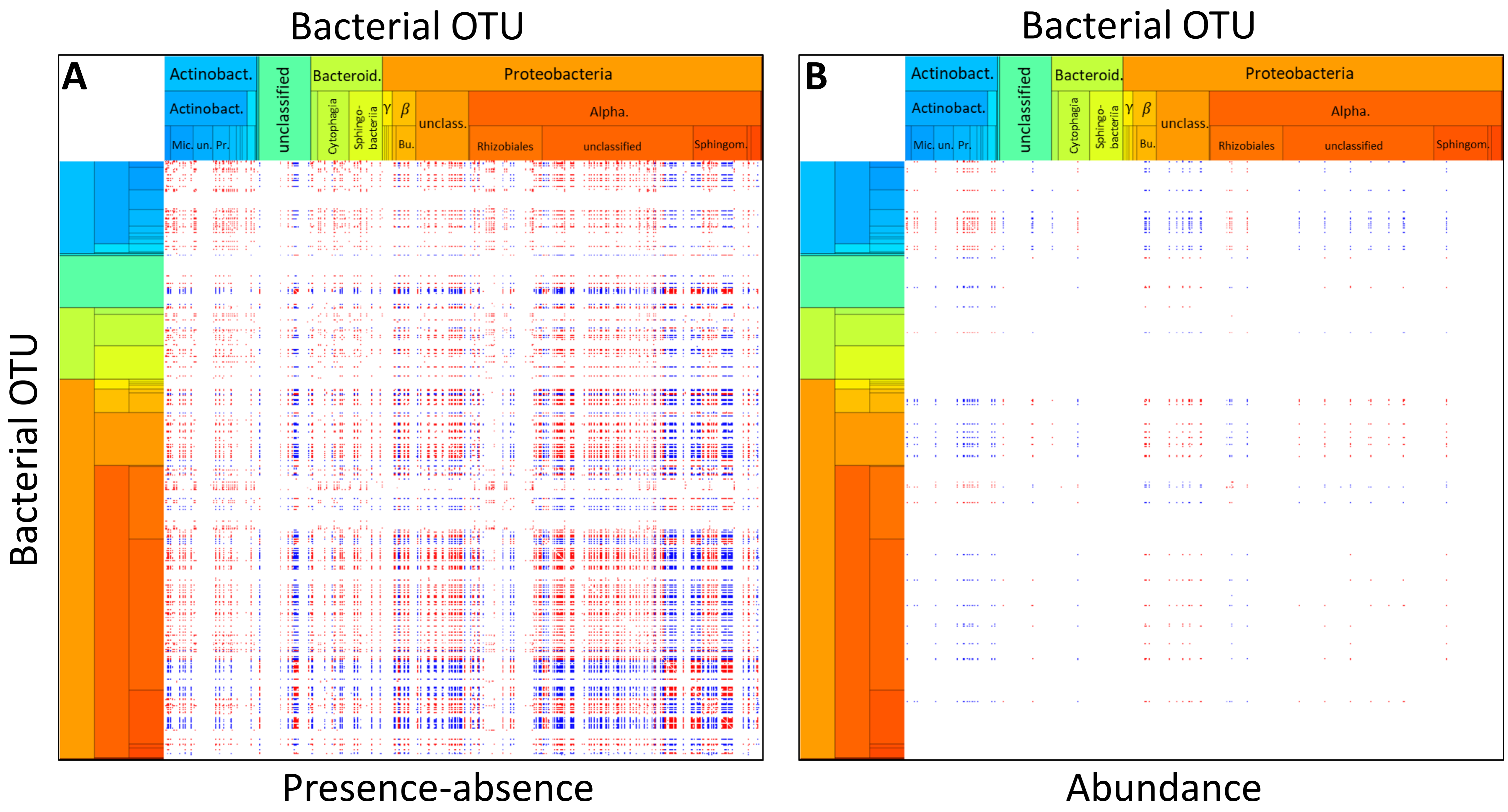

Bacterial OTU
A
Actinobact.
Actinobact.
Mic.
un.
Pr.
unclassified
Bacteroid.
Cytophagia
Sphingo-bacteriia
Proteobacteria
γ
β
Bu.
unclass.
Alpha.
Rhizobiales
unclassified
Sphingom.
Bacterial OTU
Presence-absence
Bacterial OTU
B
Actinobact.
Actinobact.
Mic.
un.
Pr.
unclassified
Bacteroid.
Cytophagia
Sphingo-bacteriia
Proteobacteria
γ
β
Bu.
unclass.
Alpha.
Rhizobiales
unclassified
Sphingom.
Abundance

Figure S6

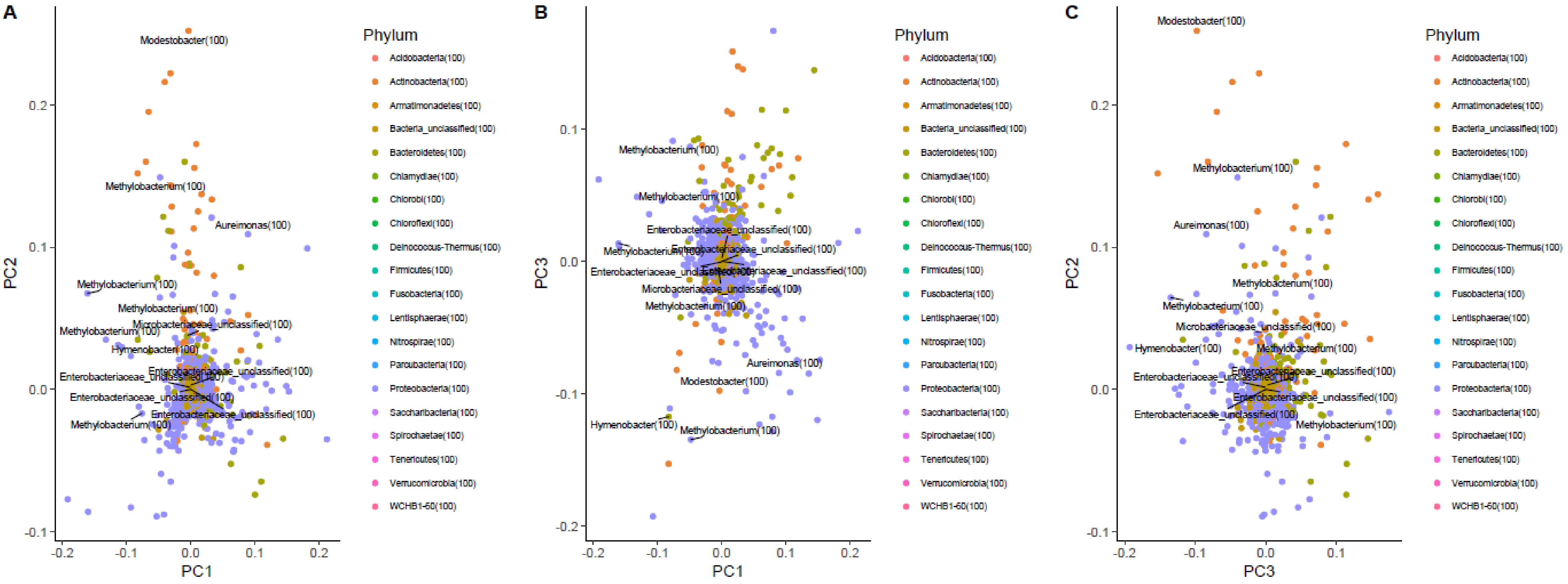
A
B
C
PC1
PC2
PC3
Phylum
Acidobacteria(100)
Actinobacteria(100)
Armatimonadetes(100)
Bacteria_unclassified(100)
Bacteroidetes(100)
Chlamydiae(100)
Chlorobi(100)
Chloroflexi(100)
Deinococcus-Thermus(100)
Firmicutes(100)
Fusobacteria(100)
Lentisphaerae(100)
Nitrospirae(100)
Parcubacteria(100)
Proteobacteria(100)
Saccharibacteria(100)
Spirochaetae(100)
Tenericutes(100)
Verrucomicrobia(100)
WCHB1-60(100)
Modestobacter(100)
Methylobacterium(100)
Aureimonas(100)
Microbacteriaceae_unclassified(100)
Hymenobacter(100)
Enterobacteriaceae_unclassified(100)

Figure S7

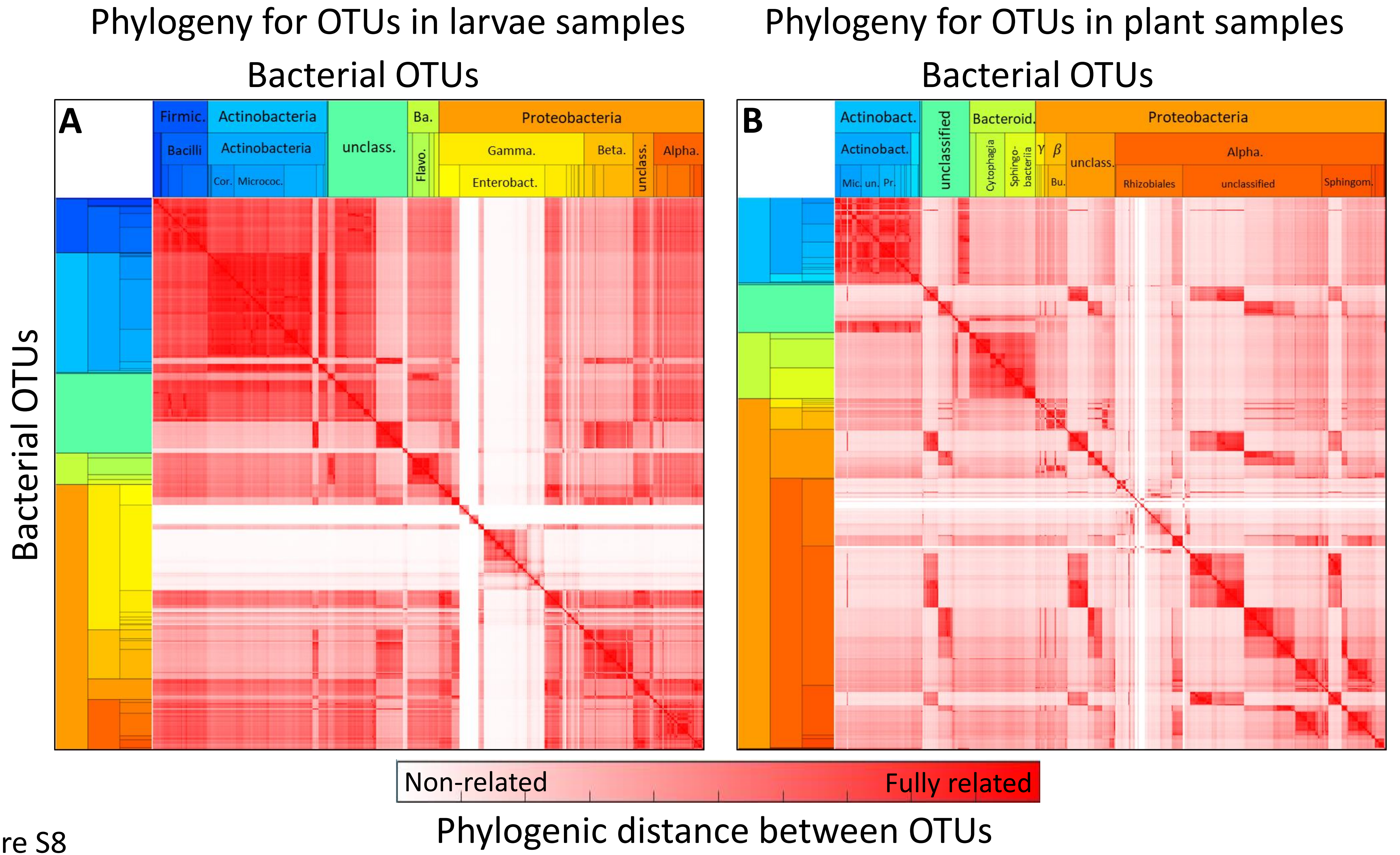
Phylogeny for OTUs in larvae samples
Bacterial OTUs
Phylogeny for OTUs in plant samples
Bacterial OTUs
A
Firmic.
Actinobacteria
unclass.
Ba.
Proteobacteria
Bacilli
Actinobacteria
Flavo.
Gamma.
Beta.
unclass.
Alpha.
Cor.
Micrococ.
Enterobact.
B
Actinobact.
unclassified
Bacteroid.
Proteobacteria
Actinobact.
Cytophagia
Sphingo-bacteriia
γ
β
unclass.
Alpha.
Mic.
un.
Pr.
Bu.
Rhizobiales
unclassified
Sphingom.
Bacterial OTUs
Non-related
Fully related
Phylogenic distance between OTUs

Figure S8
